# Pennsylvania State Veterinary Medical Association

**Published:** 1891-04

**Authors:** 


*Recording Secretary.*

---

*The Pennsylvania State Veterinary Medical Association.* The Annual meeting of the Pennsylvania State Veterinary Medical Association was held at the College of Physicians, Philadelphia March, 3, 1891. President W. S. Kooker, in the chair. The following members were present: Drs. Francis Bridge, Philadelphia; J. C. Foelker, Allentown; H. F. George, Greencastle; Robt. Gladfelter, Philadelphia; S. S. J. Harger, Philadelphia; J. R. Hart, Philadelphia; W. H. Hoskins, Philadelphia; R. S. Huidekoper, Philadelphia; W. H. Knight, Kennett Square; W. S. Kooker, Philadelphia; J. Z. Tintsman, Philadelphia; L. O. Lusson, Philadelphia; Geo. Magee, Uniontown; G. B. Rayner, Philadelphia; Thos. B. Rayner, Philadelphia; Jno. B. Rayner, Branchtown; W. E. Reinhart, Steelton; W. H. Ridge, Trevose; Chas. Schaufler, Philadelphia; D. C. Stanton, Factoryville; J. H. Timberman, Wilkesbarre; R. G. Webster, Media; W. L. Zuill, Philadelphia.

The reading of the minutes, owing to the absence of the secretary were dispensed with.

The following were nominated for officers for the ensuing year :

President, W. S. Kooker; first Vice-President, Thomas B. Rayner; second Vice-President, R. G. Webster; third Vice-President, Charles T. Goentner; Corresponing Secretary, Robert Gladfelter; Recording Secretary, Louis Olry Lusson; Treasurer, John B. Hart; Board of Trustees R. S. Huidekoper, W. H. Hoskins, T. B. Rayner, S. E. Weber, and R. H. Gladfelter.

The rules were dispensed with and the secretary was ordered to cast ballot for above officers, who were declared elected.

The following gentlemen were proposed for membership : Drs. Sturge, Koons, and Dubois, of Wilkesbarre.

Prof. Andrew Smith, of the Ontario Veterinary College, for Honorary Member, by Dr. Sallade. These names were referred to the Board of Trustees. A recess was then taken for a meeting of the Board; in the absence of two of the members, the president appointed Drs. Rayner and Webster, as temporary members.

When the meeting was reconvened the Board of Trustees reported favorably the names of Drs. Sturge, Koons, and Dubois, who were elected. The name of Dr. Andrew Smith, in the absence of Dr. Sallade was obliged to be postponed until the next meeting.

The following resolution which had been presented by Dr. Hoskins was recommended by the Board to the Association.

*Whereas*, we, the members of the Pennsylvania State Veterinary Medical Association feel a great interest in the advancement of veterinary education as a means of promoting the good standing of our profession; the improvement of our own members, and our future successors in the profession, and,

*Whereas*, we believe that a public institution, held in trust for the good of the community, should be so conducted as to be of the greatest advantage to the community at large :

*We do hereby resolve :*

That all public institutions and hospitals, which have received aid in lands and moneys, from the City and State government, should furnish free treatment to persons who from indigent circumstances, or from proper reasons are deserving of such, and, we further believe that a judicious exercise of this principle and the granting of free treatment to the animals of the proper persons is a boon to the community and can be made of inestimable value in the teaching and education of students.

*But, whereas*,

The Board of Managers of the Hospital of the Veterinary Department of the University of Pennsylvania have altered the past method of giving free treatment to animals of proper owners, and have announced free treatment of animals of *all* owners, irrespective of their means, who appeal for such, and have advertised the same through the public newspapers, and in connection with this have made a business of selling medicine, at full rates and value, in which there is no charity;

*Be it hereby resolved;* that such method is not to the advantage of the community, to the profession, or to the students who are intrusted to said hospital for education.

*Be it further resolved;* that we consider this course of the Managers of the said hospital to be prejudicial to the country's good, prejudicial to the benefit of the veterinary profession, and prejudicial to the advancement of their students, and we hereby express our regret at, and censure of their action.

The report of the Board was received and unanimously approved.

Dr. Zuill, Chairman, Committee on Sanitary Science and Police, asked to offer a verbal report, as he had found it difficult to obtain sufficient answers on the subject, from those with whom he had corresponded, to make a satisfactory one in writing. He reported that the State is free of contagious pleuro-pneumonia. Tuberculosis prevails to a great extent, and will probably increase until the legislature passes a law by which it may be properly handled. Farcy and glanders are still prevalent.

Dr. Hoskins asked if Dr. Zuill meant acute or chronic pleuro-pneumonia. Dr. Zuill answered that he had no knowledge of *any* pleuro-pneumonia in the State. The report of the committee was accepted.

Dr. Hoskins, Chairman of the Committee of Legislation made the following report:

*Mr. President and Gentlemen:*

As chairman of your Committee on Legislation I have but a brief report to make for the past six months. You will recall the suggestions made at Lancaster, that our future hope then seemed centered in such amendments to our act, as would cover the points involved in the cases we had tried and lost. To this end we have bent our efforts with the following result.

Conferring with our attorneys, Messrs. Magill and Alexander as to such changes in our bill as would cover the apparent deficiencies, they at once set to work and after some three weeks of daily consideration and consultation of all authorities on contested points of the medical act, after which our own law was largely fashioned, they reported their inability to add anything to the law at this day, that would strengthen it in anyway. They believe the law fully covers every contingency that we need and could acquire and further states the decision in Northampton County, that the six month's clause was unconstitutional, has been decided otherwise by the Supreme Court of this State, in the test case made by the physicians; they further aver, that we would have won that case in the Supreme Court, had we appealed the same. They believe that any violation of the act may be reached, and so firm is their belief on this point, that they are willing to try any case of false, post-registration, and should they lose, they would not charge one cent for their services. No amendments offered to-day could be retroactive and our only hope lies in individual action against those who have violated the act. Our hopes in this action was greatly strengthened in the decision of the Judge of Lancaster County, who pointed out the powers of our act in this direction. In this county during the past six month's there has been no registrations save those of graduates. On our records have there stood but two post-registrations of non-graduates, both by order of the court, one of which has since withdrawn from active practice. Throughout the State, as far as known, registrations save those of qualified men have ceased, and many have been denied this privilege by those entrusted with the fulfillment of the requirements of our act.

The mature decision of our attorneys assures us of the strength of our act, and I would recommend a trial or two of flagrant violators of the act during the coming six months, and if necessary, a final decision of the Supreme Court of our State. I likewise deem it important that we should test completely the legality of the decision, that Castration is not a branch of Veterinary Surgery. This question involves a principle that it is our bounden duty to sustain, for in the same catagory may fall Dentistry and it will only require a little stretching to place Veterinary Chiropody beyond the bounds of our law and the blundering, ignorant blacksmith, may ride above the law in perfect safety.

On the whole I take a sanguine view of the wholesome effect of our law. It has placed a barrier strong enough, I think, to defy breaking down at the border lines of our State, North, East, South and West. I know of no case in this county where a single violation has occurred, through the entrance from another State of any non-graduate. I am informed of several unsuccessful attempts, and in making this statement, I am reliably informed from every section of Philadelphia County. This gain is much, and promises much for the future veterinarian. We are left then to deal solely with those violaters within our own border, and I trust that ere another six month's roll by we will have additional legal light shed upon our law, and thus be better entrenched for future action. Respectfully submitted,

W. Horace Hoskins, *Chairmau.*

Dr. Hoskins added.

"We should decide whether castration was a branch of veterinary surgery, and that the incoming committee should forcibly act upon this subject. Some expense had been incurred by this committee through their attorneys, Mr. J. G. Johnson and Mr. F. Magill, and Dr. Huidekoper said that Mr. Johnson offered his services gratis. A vote of thanks was extended to Mr. Johnson. It was moved and seconded that the above report be accepted."

*Report oj the Committee on Intelligence and Education.*—Mr. President: This committee having made a full report upon its subject at the last meeting of the association in September last, finds but little to add to it. The local associations of the State. at Philadelphia, in Franklin County, and that of the north western part of the State, held in conjunction with members of Ohio and New York States, have held regular and interesting meetings. We fear that no very active work has been done since our last meeting to stimulate the formation of new local societies, although we hear of two or three that are on the way to organization; we think it would be well to have a thorough investigation of the wordings of the codes of ethics and when any such code is adopted, it should treat in a perfectly distinct way, of general principles only, and not be complicated with details, which can be made the basis for quibling and waste of time on the part of those who wish to make trouble. Let the association be jealous of their membership and careful to the last degree in the character of those who they receive into membership, and there will then be less ground for charges and disputes

We can learn of no changes of importance in the curriculum of the veterinary schools.

The publication of The Journal of Comparative Medicine And Veterinary Archives, has been removed from Philadelphia to New York, as a matter of greater convenience to its editors, but its pages will continue, as they have been, freely open to the pens of the Veterinarians of Pennsylvania.

The meeting of the United States Veterinary Medical Association, held at Chicago, proved to be the greatest meeting, in both the numbers that attended and in ths scientific character of the papers and reports presented, that has ever been held in this country. It certainly furnished a great stimulant to the organizations throughout the country and we are glad to see that the new associations are organizing with a view to become an elementary part of the national association.

The present meeting at the College of Physicians at Philadelphia, the building of the greatest medical society in the United States, is we consider, an important step in the public recognition which it gives to the veterinary profession as a component part of the great science of medicine.

We recommend that the Readings of this Association, including essays and reports of committees be published in pamphlet form, not only for the members, but for circulation to reputable practitioners throughout the State. We beg to call your attention to the organization of an association of dairymen which has been acheived by Dr. Sallade. It meets once a month. Some of the objects in view are: 1st. Mutual protection to its members. All animals are branded. 2d. The purification of meat, milk and other dairy products. 3d. To educate and create friendly feelings towards each other and to the veterinary profession in particular. Insurance of animals is carried on. All animals are appraised after death. Over $800 has been paid to owners for losses sustained from tuberculosis and other causes. A yearly pic-nic and ball of the "Bovine Association," aids its funds. The Veterinarian receives a yearly salary as lecturer to the association. The result has been a very friendly feeling in the County towards the veterinary profession. Very respectfully submitted,

Rush S. Huidekoper, *Chairman.*
H. G. Pagat.
James A. Sallade.

Dr. Hoskins announced that the next annual meeting of the United States Veterinary Medical Association would be held in September, at Washington, D. C., and further urged that as many as possible would join the United States Association.

Dr. William L. Zuill then read a paper on "Indigestion in the Horse, Gastric and Intestinal."

In the debate on Dr. Zuill's paper, Dr. Hoskins asked if the essayist advocated exercise in all cases of gastric indigestion. In reply Dr. Zuill stated that where exercise was at all possible that he thought it advisable. Dr. Ridge thought that bran and cracked corn mixed were most frequent causes of gastric indigestion, because this food was swallowed without masticating. Dr. Michener inquired of the essayist what his idea was in giving emetics to the horse. Dr. Zuill stated that emetics were not given to cause emesis but to contract the mucous membrane and force the food to the lower bowel. The essayist was accorded a vote of thanks for his valuable paper.

Dr. J. Curtis Michener then presented a paper on Meningitis, (see page 157.)

Debate on Dr. Michener's paper. Dr. Hoskins complimented Dr. Michener for his most elaborate and complete paper, upon a subject so interesting to the veterinarian and recommended that it be printed for circulation. Dr. Huidekoper stated that THE JOURNAL OF COMPARATIVE MEDICINE AND VETERINARY ARCHIVES would furnish the association with copies for its members. Dr. Harger thought 500 copies should be printed for circulation. Dr. Hoskins amended his motion by making it 1,000, which was agreed upon. Dr. Huidekoper asked the essayist if decomposed corn stalks caused any different kind of symptoms from those caused by other decomposing material. Dr. Michener said he did not recognize any difference, that this disease was liable to give any train of symptoms whatever. Dr. Rayner cited some cases where the animals had occupied a barn yard containing decomposing corn stalks. Dr. Huidekoper cited a case where the drenching of a building, after a fire, was followed by the disease, all the proprietor's horses (5) died, a sixth horse a boarder, fed from without, was not attacked. He inquired of Dr. Michener, whether he had found a low temperature favored the disease. Dr. Michener replied that it occurred at all seasons, most prevalent in August and September, and usually began to appear after harvest. Dr. Michener was accorded a vote of thanks by the association.

An application for membership was made by Dr. H. C. Miller, of Jenkintown, Pa., vouched for by Dr. Chas. Schauffler, of Philadelphia, was handed to the secretary.

Dr. Ridge, of Trevose, Pa., cited a case of simulating Dourine, (see page 171).

The Treasurer submitted the following report for the year ending March 3, 1891.:

| | | |
|---|---|---|
| March 4, 1890. | Balance in treasury.................. | $ 11.76 |
| Sept. 2, 1890. | Received as dues and membership fees. | 132.00 |
| | | $143.76 |
| Report amount paid for printing, postage and legislative com., for year ending March 3, 1891.................. | | $83.62 |
| Leaving balance in treasury of.... .................... | | 60.14 |
| Report delinquent members fourteen... .............. | | |
| Indebtedness of delinquent members.............. .. | | $97.00 |

J. R. HART, *Treasurer.*

On motion it was resolved by the association that the recording secretary should render statements each year of the indebtedness of its members.

The following committees were appointed by the president :

Committee on Legislation. Dr. Hoskins Chairman, Drs. R. S. Huidekoper, Francis Bridge, Robert Gladfelter, and Jas. Rayner.

Committee on Intelligence and education. Dr. Huidekoper Chairman, Drs. J. W. Sallade, H. A. Paget.

Committee on Sanitary Science and Police. Dr. Zuill Chairman, Drs. Francis Bridge, J. B. Rayner, J. H. Timberman, J. C. Michener, Geo. Magee and J. L. Bradley.

On vote of the association it was agreed to hold the September meeting at Wilkesbarre, Pa. Bills were presented by the corresponding secretary for printing, stamps, spencerian writing, (on certificates) etc., for $17.50. The treasurer was instructed to draw an order for the full amount for the above bill.

The new officers were seated and the meeting adjourned at 5.15 P. M.

L. O. LUSSON, *Secretary*.

